# Buffalo Academy of Medicine

**Published:** 1911-11

**Authors:** 


### Buffalo Academy of Medicine

November 9, 1911.—Surgical Section, Construction and Mechanism of Moveable Joints, Dr. Gwilym G. Davis, Philadelphia.

November 14.—Medical Section, Normal and Pathologic Physiology of the Hypophysis, Dr. Joseph L. Miller of Chicago.

November 21.—Obstetric and Gynecologic Section, Merits of the Cautery in Radical Operation for Carcinoma of the Cervix, Dr. X. O. Werder, of Pittsburg.

November 28.—Pathologic Section, Present Status of Syphilis Research, Dr. Hideye Noguchi, of New York.

